# Ultrafast laser induced local magnetization dynamics in Heusler compounds

**DOI:** 10.1038/srep38911

**Published:** 2016-12-14

**Authors:** P. Elliott, T. Müller, J. K. Dewhurst, S. Sharma, E. K. U. Gross

**Affiliations:** 1 Max Planck Institute of Microstructure Physics, Weinberg 2, D-06120 Halle, Germany

## Abstract

The overarching goal of the field of femtomagnetism is to control, via laser light, the magnetic structure of matter on a femtosecond time scale. The temporal limits to the light-magnetism interaction are governed by the fact that the electron spin interacts indirectly with light, with current studies showing a laser induced global loss in the magnetic moment on a time scale of the order of a few 100 s of femtoseconds. In this work, by means of *ab-initio* calculations, we show that more complex magnetic materials - we use the example of the Heusler and half-Heusler alloys - allow for purely optical excitations to cause a significant change in the local moments on the order of 5 fs. This, being purely optical in nature, represents the ultimate mechanism for the short time scale manipulation of spins. Furthermore, we demonstrate that qualitative behaviour of this rich magnetic response to laser light can be deduced from the ground-state spectrum, thus providing a route to tailoring the response of some complex magnetic materials, like the Heuslers, to laser light by the well established methods for material design from ground-state calculations.

The ultimate goal of femtomagnetism^1^,^2^ is to manipulate, on very short time scales, the spin structure of matter by intense laser light. This search for a femtosecond control of matter is driven both by fundamental physical interest, as well as by the promise of a consequent revolution in the speed of magnetic storage devices^3^. Central to the interaction of laser light with matter are the time scales on which the multitude of induced excitations occur^4^,^5^. For *t* < ≈30 fs light-matter coupling is dominated by the purely electronic degrees of freedom that, at longer time scales, is followed by dissipation of the excited electronic system via electron-phonon scattering, spin diffusion, and radiative processes. Most promising for spin control is the pre-dissipative coherent regime in which only the electronic degrees of freedom of the system are excited.

Both experiment and theory universally report an initial decrease in the global moment subsequent to a pulse of laser light^6^,^7^,^8^,^9^,^10^,^11^,^12^,^13^,^14^,^15^,^16^,^17^,^18^ for the elemental magnetics Fe, Ni, and Co. In this work we demonstrate that the *local* moments of complex ferromagnetic and anti-ferromagnetic materials exhibit, hidden in their sub-lattice structure, a much richer response than a simple reduction of moment (see Fig. 1). This provides the first indication that the coherent regime possesses the richness of magnetic response that might presage the long sought after control of magnetic structure by light.

Previous theoretical work has ascribed the initial moment loss in elemental magnets Fe, Co, and Ni to a variety of mechanisms: spin-orbit induced spin flips^19^,^20^,^21^, spin currents^16^,^22^,^23^,^24^, and Elliot-Yaffet spin flip scattering^17^,^25^,^26^,^27^ being the most prominent. All these processes indirectly couple light to spins, and it is the indirect nature of this coupling that dictates the temporal duration of this initial demagnetization. Here we study not elemental magnetic but complex magnetic materials represented by a diverse range of Heuslers^28^: NiMnSb, Co_2_MnSi, Mn_3_Ga, Ni_2_MnGa, and Co_2_FeSi. The key feature of the Heusler alloys from the point of view of their response to laser light^18^,^29^,^30^, and what differentiates them from the elemental magnets, is the structure of their spin and sub-lattice projected ground-state spectrum. As we will show, this allows for optical excitations to drive a spin and charge current between sub-lattices, leading to an increase of local moment on one sub-lattice and a corresponding decrease on the other. This has two important consequences: (i) it reduces the time scale on which the magnetic response to light occurs and, as we will show, (ii) the qualitative form of this response may be deduced from the ground-state spectrum. In regard to the first of these two points we should stress that while the time scale of the response to a laser pulse obviously depends on the pulse parameters, the manipulation of magnetic structures by optical transitions alone is the fastest possible response of magnetic matter to light.

## Results and Discussion

**Figure 1:**
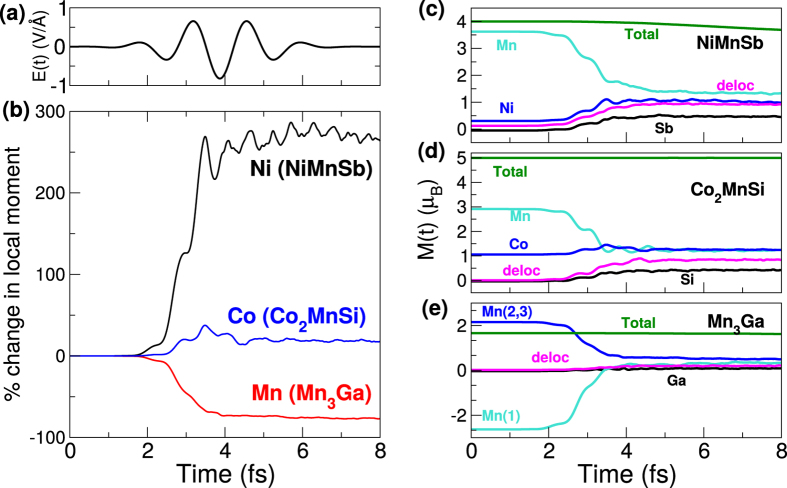
(**a**) The electric field, *E*(*t*), of the applied laser pulse in V/Å. (**b**) The dynamics of the *relative* change in the local magnetic moment on Ni, Mn(2,3) and Co atoms for Heusler compounds NiMnSb, Mn_3_Ga, and Co_2_MnSi respectively. The dynamics of the *absolute* local moment in *μ*_B_ on each atom and of the delocalized moment for the Heusler compounds (**c**) NiMnSb, (**d**) Co_2_MnSi and (**e**) Mn_3_Ga.

As representative examples of the Heusler compounds^31^,^32^,^33^,^34^,^35^,^36^,^37^,^38^,^39^,^40^,^41^,^42^ we will first focus on NiMnSb, Co_2_MnSi, and Mn_3_Ga. The first two of these materials are ferro-magnetically coupled while Mn_3_Ga exhibits an anti-ferromagnetic coupling between the Mn sub-lattices; detailed structural and ground-state properties may be found in Table 1. Under the influence of an external laser field, the form of which is presented in the Fig. 1(a), the relative local moment (i.e., the quantity (*M*(*t*) − *M*(*t* = 0))/*M*(*t* = 0)) changes dramatically; Ni in NiMnSb is seen to exhibit a ~300% increase, Mn(2,3) atoms (these are the two Mn atoms at the 4d crystal sites) in Mn_3_Ga show a 100% decrease, while in contrast for Co in Co_2_MnSi the change in the local moment is negligible. These materials share one universal characteristic which may be seen in Fig. 1(c,d and e): *the laser pulse induces transfer of local moment from one sub-lattice to another with very little change in the global moment*. As in the elemental magnets^19^, at longer time scales (≥10 fs) spin-flip scattering processes begin to dominate and drive a global demagnetization. What we will focus on here is the richer ultrafast regime in which a sub-lattice differentiated response to the laser pulse is seen, and in which both increase and decrease in local moments may occur. These results pose several questions which we will devote the rest of the paper to answering: (i) What causes this ultra-fast and dramatic change in the local moment? (ii) Why do the details of the changes in magnetic structure differ between the three materials? (iii) How significantly does the spin dynamics depend upon the choice of the laser pulse parameters?

### A case study of 5 Heuslers

**Figure 2:**
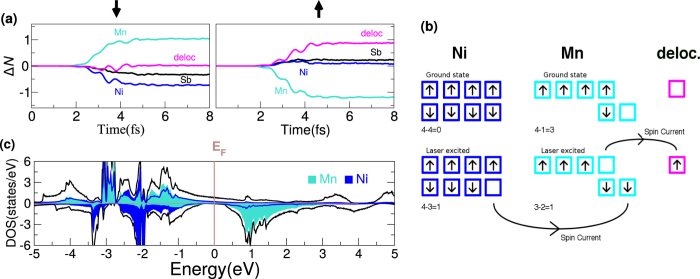
(**a**) The time-dependent change in the spin-down (left) and spin-up (right) electrons on each atom (and of the delocalized electrons) relative to the ground-state for NiMnSb. (**b**) Schematic of the spin up and spin down occupations for Ni, Mn and the delocalized electrons (deloc), demonstrating how optical excitations cause an increase in the local moment on Ni and a decrease on the Mn atom. In the schematic the occupations are rounded to the nearest integer for simplicity. (**c**) The spin and atom resolved density of states (in states/eV).

In order to answer the first of these questions we take a closer look at the case of NiMnSb. In Fig. 2(a) are plotted the dynamics of the spin occupation as a function of time:


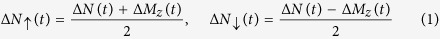


where Δ*N*(*t*) = *N*(*t*) − *N*(*t* = 0) is the change in the local charge, at time *t*, as compared to the initial charge, and similarly the change in the *z*-projected local moment is Δ*M*_*z*_(*t*) = *M*_*z*_(*t*) − *M*_*z*_(*t* = 0). This definition is, strictly speaking, valid only for collinear magnetic systems. However, as the magnetic structure of this material remains almost perfectly collinear for the first ~10 fs, despite the presence of spin-orbit coupling (SOC) and the external laser field, both of which allow for interatomic non-collinearity, this definition can safely be deployed. In what follows we will, as per the usual convention for ferromagnetic materials, label the minority channel as spin down. It is clear from Fig. 2(a) that these minority electrons are transfered from the Ni to the Mn sub-lattice, and this inter- sub-lattice minority spin current drives an increase in the Ni sub-lattice moment and a corresponding decrease in Mn sub-lattice moment with almost no change in the global moment. This constancy of the global moment implies that there are no spin flip processes and optical excitations alone are responsible for this spin transfer. An additional decrease in the moment on the Mn sub-lattice occurs due to the excitation of Mn majority spin electrons to high-lying delocalized states. What remains to be addressed is the question as to why light induces such a spin transfer. This may be answered by examination of the ground-state DOS which we present in Fig. 2(c) and from which we can immediately see that the minority channel is dominated by Ni *d*-states below the Fermi energy and Mn *d*-states above it. A simple availability of states argument then ensures that the laser pulse excites occupied minority Ni electrons to unoccupied minority Mn *d*-states. Since this charge flow is spin selective, i.e. is dominated by the minority spin electrons, it is equivalent to a flow of minority spin current between the two sub-lattices. The essence of this process is summarized schematically in Fig. 2(b): Loss of minority spins on the Ni sub-lattice, which has an almost fully occupied *d*-band, can only lead to an increase in the local moment while, on the other hand, the Mn *d*-band is almost exactly half filled (hence the large Mn sublattice moment) and any gain in the minority spins can only cause a loss in the local moment.

**Figure 3:**
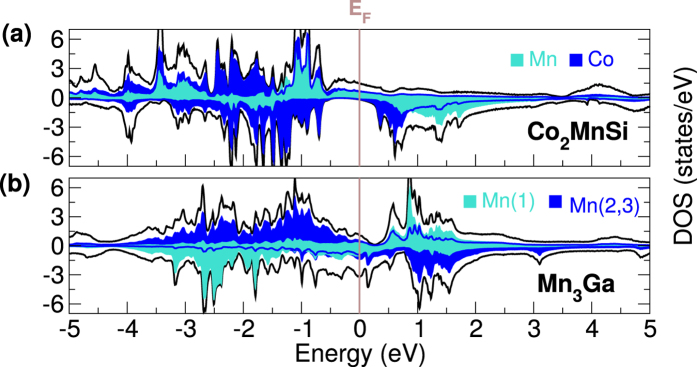
The spin resolved total density of states (in states/eV) for (**a**) Co_2_MnSi and (**b**) Mn_3_Ga. In each case the contribution of the Co and Mn *d*-states are given by the filled area.

The central role played by the ground-state density of states of NiMnSb in the *type* of spin current induced by the laser pulse suggests that this may hold the key to understanding the different responses of the other Heusler alloys. To confirm this we now examine the ground-state DOS for Co_2_MnSi and Mn_3_Ga (see Fig. 3). The DOS for Co_2_MnSi is very different from NiMnSb in that the states above the ground-state Fermi level exhibit a significant projection on both magnetic sublattices and thus two distinct optical excitations are possible. Namely, the laser may excite occupied Co minority *d* states to either (i) unoccupied Co minority *d* states or (ii) unoccupied Mn minority *d* states. Only the former of these excitations will generate an inter-sublattice spin current and, as may be seen from Fig. 1, the largely unchanged Co moment under excitation by the laser pulse indicates that it is the former excitation which dominates the physics in this material. Note that, as in the case of NiMnSb, a certain proportion of the majority Mn states are excited into high-lying delocalized states, which results in the small decrease in the Mn sub-lattice moment that can be observed in Fig. 1. This difference in behaviour between Co_2_MnSi and NiMnSb may already have been seen experimentally^29^,^30^.

The DOS for Mn_3_Ga presents, in the spin up channel, exactly the same qualitative feature as found in the NiMnSb spin down channel: either side of the Fermi level the DOS is dominated by states from distinct sublattices. In this case the occupied states are dominated by the Mn(2,3) sublattices (the 4d crystal sites) and the unoccupied by the Mn(1) sublattice (the 2b crystal site). There is, as in the case of NiMnSb and in contrast to Co_2_MnSi, therefore only one type of optical excitation which the laser may induce: from Mn(2,3) to Mn(1) dominated states. The laser pulse will therefore excite spin up electrons from Mn(2,3) to Mn(1) states. However, the crucial difference from NiMnSb is that these sub-lattices are anti-ferromagnetically coupled and, as a consequence, spin up is majority on Mn(2,3) but minority on Mn(1). The laser induced spin up current flow therefore leads to a decrease in *both* the Mn(1) and Mn(2,3) sub-lattices, exactly as seen in Fig. 1(b). Interestingly, we find that Mn(1) atom flips its spin at ~4 fs and Mn_3_Ga becomes ferro-magnetically coupled. The moment on each Mn site is, however, very small beyond 4 fs (0.48 *μ*_B_ on Mn(2,3) and 0.37 *μ*_B_ on Mn(1)) and it remains to be seen if such a change in magnetic order can be experimentally observed. It is also worth mentioning that this change from anti-ferromagnetic to ferromagnetic order in Mn_3_Ga is something which cannot be anticipated from the ground-state DOS, and one requires TDDFT calculations to see this.

This analysis therefore leads us to the following conclusion: in Heusler materials the laser pulse induced early time spin dynamics is dominated by inter-sublattice spin and charge currents that are induced by optical excitations. Qualitative features of the response to the laser pulse, in particular whether there will be an increase (NiMnSb), decrease (Mn_3_Ga), or very little change (Co_2_MnSi) in the local moments, may be inferred from the qualitative form of ground-state density of states. The examples presented here would, furthermore, indicate that the spin dynamics response of the Heusler materials can broadly be divided into two classes: (a) materials in which the ground-state Fermi energy lies between *d*-states from different magnetic sub-lattices and (b) materials in which the DOS above the Fermi level is composed of states from both magnetic sublattices. The former will show strong laser induced flow of inter-sublattice spin current, which will be largely suppressed in the latter.

**Figure 4:**
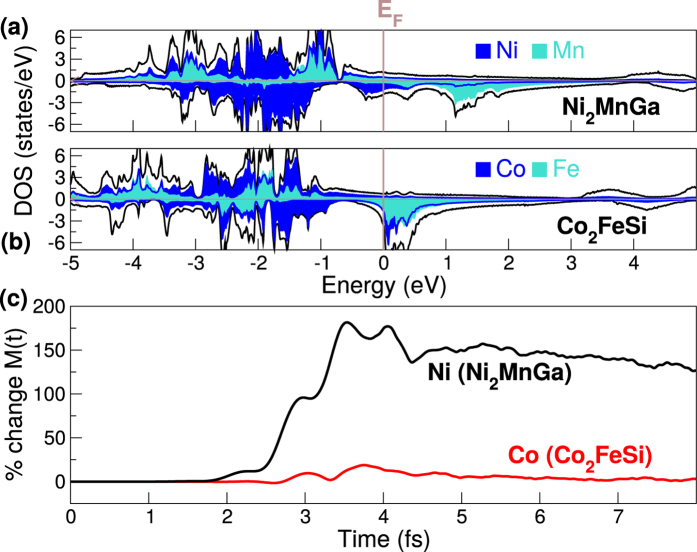
The spin resolved density of states for (**a**) Ni_2_MnGa and (**b**) Co_2_FeSi, the contribution of the Ni, Co and Mn *d*-states are given by the filled area. (**c**) The percentage change of the relative local moment of Ni for the compound Ni_2_MnGa and Co in the compound Co_2_FeSi.

To probe the validity of this classification of the Heuslers we have performed calculations of the laser induced change in magnetic structure for an extended set of materials: Co_2_FeGa, Co_2_FeIn, Co_2_FeSn, Co_2_NiAl, Co_2_NiGe, Co_2_NiGa, Co_2_NiIn, Co_2_NiSn, Co_2_MnAl, Co_2_MnGe, Co_2_FeAl, Co_2_PtGa, Co_2_PtSn, Fe_2_CoGe, CoFeGe, CoMnSb, Fe_2_CoSn, Fe_2_NiAl, and Ni_2_MnGa. All these materials possess a DOS that is either of the NiMnSb type or the Co_2_MnSi type. For the former class of materials we find in each case a laser induced inter-sublattice current flow, leading to significant changes in the local moments, while for the latter class the change in local moment is always very small. This latter result is rather striking: when the ground-state DOS presents the possibility of optical excitations that will either generate or not generate an inter-sublattice current, the latter choice is always preferred. In Fig. 4 we present two more examples, from this set, of these two classes of Heuslers: Ni_2_MnGa and Co_2_FeSi. The DOS for Ni_2_MnGa belongs to the first class (see Fig. 4(a)); above the Fermi level the DOS is dominated by Mn-*d* and below by Ni-*d* states. Optical excitations then lead to an increase in the local moment on the Ni site (see Fig. 4(c)). Co_2_FeSi on the other hand belongs to the second class (see Fig. 4(b)) and only a very small change in the local moment on the Co site occurs. These results for a wide range of Heuslers demonstrate that the qualitative form of the ground-state DOS can determine the nature of the short time laser induced spin dynamics in these materials. All details of the magnetization dynamics beyond the broad outline provided by the classification scheme can, of course, only be obtained from full TDDFT calculations.

### Time resolved density of states

**Figure 5:**
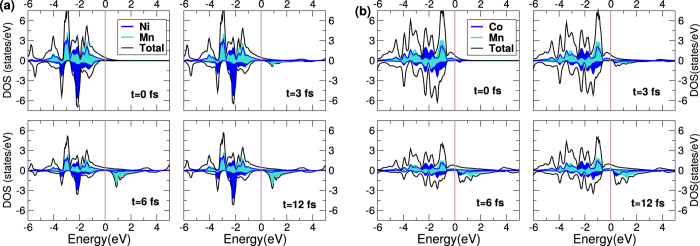
Time resolved occupied density of states (in states/eV), for (**a**) NiMnSb and (**b**) Co_2_MnSi. Snap-shots at four different times (0, 3, 6 and 12 fs) are shown.

The ultimate proof of the ground-state DOS interpretation, however, must rest on actual time-resolved DOS. In Fig. 5 are plotted a time-resolved DOS for representative materials of both classes of Heuslers: NiMnSb and Co_2_MnSi. In both materials the spin (and charge) dynamics is dominated by the minority (down spin). However, in the case of NiMnSb electrons are excited from Ni *d*-states (dark blue) to Mn *d*-states (light blue) as a function of time; a comparison of the DOS at 0 and 6 fs reveals a significant loss in Ni *d*-electrons (at around −2 eV) and a corresponding gain in Mn *d* electrons (at around 1 eV). It is interesting to note that the minority DOS at 12 fs shows a net gain in Ni *d*-electrons (again at −2eV). This however, is caused by spin-orbit induced spin-flips which results not only in a local demagnetisation of Ni sub-lattice but, as spin is not conserved, also in a global loss of moment.

In contrast, for the case of Co_2_MnSi a comparison of DOS at 0 fs and 6 fs reveals that the loss of Co *d*-electrons at around −1.5 eV is almost equal to the gain in Co *d*-electrons at 1 eV. This then leads to almost no change in the Co local moment. On the other hand, loss in Mn *d*-electrons around −2 eV is much larger than the gain in Mn *d*-states at 2 eV, as some of the Mn *d*-electrons are excited to higher lying delocalised states. This process then causes a net loss in the Mn local moment. It is also important to mention that spin-orbit induced flips are temporally separated from optically driven excitations in Co_2_MnSi by about 20 fs and thus no net increase in minority spin electrons is seen at 12 fs.

This behaviour of time-resolved DOS indicates that, in concordance with the analysis provided in the previous sections, the ground-state spectrum provides a very good guide to the type of laser induced excitations in this class of materials. This is remarkable when one considers that the pulses used in this work are very *intense* and all sorts of non-linear processes are allowed. This indicates that this class of materials (Heuslers) behave very differently from elemental solids where higher order process and spin-orbit play the major role in the initial laser driven spin dynamics.

### Dependence on laser pulse parameters

**Figure 6:**
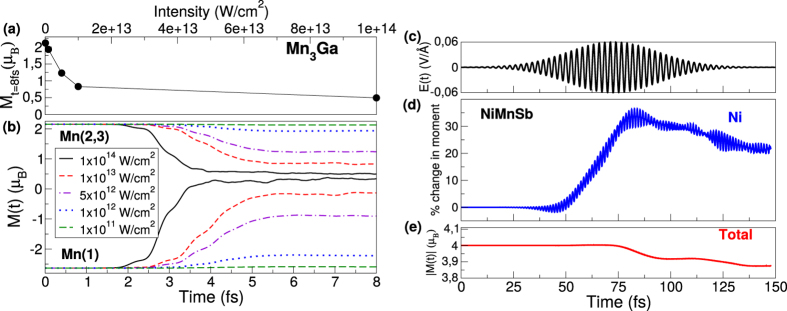
(**a**) Value of the plateau moment (in *μ*_*B*_) on the Mn(2,3) atoms as a function of the intensity of the applied laser field (in W/cm^2^). (**b**) The dynamics of the local moment on the Mn(1) and Mn(2,3) atoms in Mn_3_Ga for several different laser intensities (the laser field is the same as in Fig. 1). (**c**) The electric field of the applied laser pulse with *ω* = 1.55 eV (*λ* = 800 nm), FWHM = 50 fs, and a fluence of 10.1 mJ/cm^2^ giving a peak intensity of 5.4 × 10^11^ W/cm^2^. (**d**) The percentage change of the local moment on Ni atom in NiMnSb. (**e**) The dynamics of the absolute value of the total moment (in *μ*_*B*_).

We now turn to the final of the three questions asked at the outset of this paper: how important are the laser pulse parameters in the qualitative response of the material? This question is germane as (a) the *duration* (FWHM) of the laser pulses used so far in this work are much shorter than those commonly found in present day experiments and (b) the *intensity* of the applied laser pulses are very high compared to common experiments. To that end we present in Fig. 6(b) the time dependence of the Mn(1) and Mn(2,3) local moments for several different laser intensities, from the high intensity test pulses used in the previous section down to intensities commonly found in experiment. As may be seen, changing the intensity of the laser pulse results essentially only in a scaling of the time dependent *M*(*t*) plots. The change in the magnetic order, i.e. the spin flip of the Mn(1) sublattice, is only seen for laser pulses with very high intensity (10^14^ W/cm^2^) in which a large fraction of electrons are excited above the Fermi level. For low intensity pulses the Mn atoms remain always anti-ferromagnetically coupled. It is also interesting to note that after the initial all optical dynamics the moment shows a plateau, from ~5–15 fs, beyond which spin-orbit induced spin-flips lead to a global demagnetization. The value of the moment at this plateau (Fig. 6(a)) shows a highly non-linear behaviour as a function of the pulse intensity.

The final laser pulse parameter that we must explore is the pulse duration and to that end we apply to NiMnSb a pulse with parameters commonly used in experiments (shown in the top panel of Fig. 6(c)). In Fig. 6(d) the percentage change in the local moment of Ni is given, while Fig. 6(e) shows dynamics of the total moment. A comparison of the time dependence of the local Ni and global NiMnSb moments reveals two distinct physical regimes. An early time regime dominated by optical excitations in which only the local moment shows substantial change, followed at longer times (*t* > ≈80 fs) by spin flip processes which drive a reduction in both local and global moments. The physics of optical transitions driving changes in the local moments therefore can be found for all laser pulses: both the test pulses we have used earlier in this work, as well as pulses with intensities and wave-packet envelopes that correspond to those commonly used in experiments.

## Conclusions

The main conclusion from this work is that complex magnetic materials exhibit an ultrafast response to light governed by optical excitations alone. These optical excitations drive inter-sub-lattice spin currents that result in a redistribution of spin between sub-lattices but no change in the global moment of the material. At longer time scales spin flip scattering processes can occur with a consequent reduction in the global moment, for pulse parameters commonly used in experiment these two regimes can be easily distinguished. Any experimental probe sensitive to a local spin structure will therefore be able to measure such effects.

The change of magnetic matter by laser induced optical excitations represents both the ultrafast limit of femtomagnetism, as well as a richer response to laser light than is has hitherto been seen in magnetic materials. Most importantly, as the change in magnetic structure is through optical excitations, the qualitative form of this change may be inferred from the ground-state spectrum, a situation that will likely hold for all materials with two (or more) magnetic sub-lattices. A remarkable corollary of this immediately follows: material design for a specific response to laser light may, to a substantial extent, be guided by the ground-state spectrum. As the material design of ground-state electronic structure is a very well established field, this opens a new avenue in the field of femtomagnetism.

## Methodology

To study the spin and charge dynamics in these materials under the influence of ultrafast laser pulses, we have used the *ab-initio* method of time-dependent density functional theory (TDDFT). For the present work we have employed the non-collinear spin-dependent version of TDDFT where the time-dependent Kohn-Sham (KS) orbitals are treated as Pauli spinors determined by the equations:


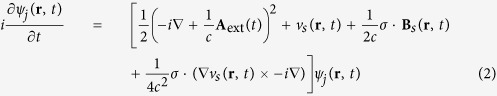


where **A**_ext_(*t*) is a vector potential representing the applied laser field, and *σ* are the Pauli matrices. The KS effective potential *v*_*s*_(**r**, *t*) = *v*_ext_(**r**, *t*) + *v*_H_(**r**, *t*) + *v*_xc_(**r**, *t*) is decomposed into the external potential *v*_ext_, the classical electrostatic Hartree potential *v*_H_ and the exchange-correlation (XC) potential *v*_xc_. Similarly the KS magnetic field is written as **B**_*s*_(**r**, *t*) = **B**_ext_(*t*) + **B**_xc_(**r**, *t*) where **B**_ext_(*t*) is the magnetic field of the applied laser pulse plus possibly an additional magnetic field and **B**_xc_(**r**, *t*) is the XC magnetic field. The final term of Eq. 2 is the spin-orbit coupling term. Since the wavelength of the applied laser in the present work is much greater than the size of a unit cell we apply the dipole approximation and hence disregard the spatial dependence of the vector potential. For more details on TDDFT we refer the reader to refs 43, 44, 45 and 46, and in particular for its application to spin dynamics refs 19, 47, 48 and 49.

With the *ψ*_*j*_’s in hand a time-resolved DOS, shown in Fig. 5, can be calculated using the following:


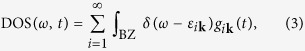


with


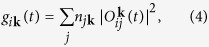


where *n*_*j***k**_ is the occupation number of the *j*^th^ time-evolving orbital and


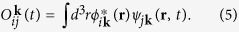


Here *ϕ*_*i*_ are the ground-state Kohn-Sham orbitals. In absence of any time-dependent perturbation 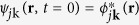 and Eq. 3 gives the ground-state DOS. Equation (3) is a simple working definition of time-resolved DOS created in order to provide insight into the processes at play as a function of time.

Early time spin-dynamics in Heuslers is governed by the flow of spin current. The spin current density tensor, 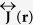, is defined as the expectation value of the operator 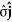 where *σ* are the Pauli spin matrices and 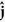 is the usual electronic current density operator (including both paramagnetic and diamagnetic contributions). In order to understand the nature of this tensor one can look at the equation of motion of the magnetization density:


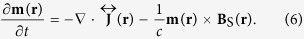


Here 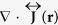 is a vector as when written component wise it looks like 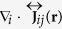. In the absence of an external magnetic field and when using a locally collinear XC functional, like adiabatic local spin density approximation (ALSDA), the second term on the right is identically zero and this equation reduces to a continuity equation for the magnetization density. This implies that the spin currents transport magnetization from one part of the system to another.

To perform our calculations we use the highly accurate full potential linearized augmented-plane-wave method with 2-component spinors, as implemented in the ELK^50^ code. In all calculations a regular mesh in **k**-space of 8 × 8 × 8 grid points was used and a time step of Δ*t* = 0.05 au is employed for the time-propagation algorithm^19^. The final magnetization value for each atom is converged with these parameters. However, there are small oscillations around this final value, such as those seen in Fig. 1(b). These oscillations are numerical and get damped as you increase the number of **k**-points. In contrast to this the rapid oscillations seen in Fig. 6(d) are due to the electrons moving back and forth with the frequency of the electric field (as well as higher harmonics). For mathematical reasons, which can be easily verified, integration of a quantity within a sphere around the atom (which is done to obtain the local moment) leads to a doubling of the frequency of any oscillation and hence the frequency in Fig. 6(d) is twice that in Fig. 6(c). The laser field applied in all cases (unless otherwise stated) is shown in the upper panel of Fig. 1(a). This pulse has a frequency *ω* = 2.72 eV, a FWHM of 2.42 fs, and fluence of 93.5 mJ/cm^2^, giving a peak intensity of 1 × 10^14^ W/cm^2^. The purpose of this ultrashort pulse was primarily to disentangle the optical excitation process from any subsequent dynamics such as spin-orbit mediated demagnetization. We also perform calculations with much weaker (intensities as low as 1 × 10^11^) and longer pulses (FWHM = 50 fs, *ω* = 1.55 eV (*λ* = 800 nm) and a fluence of 10.1 mJ/cm^2^ giving a peak intensity of 5.4 × 10^11^ W/cm^2^), which are closer to the ones routinely used in current experimental work.

## Additional Information

**How to cite this article**: Elliott, P. *et al*. Ultrafast laser induced local magnetization dynamics in Heusler compounds. *Sci. Rep.* **6**, 38911; doi: 10.1038/srep38911 (2016).

**Publisher's note:** Springer Nature remains neutral with regard to jurisdictional claims in published maps and institutional affiliations.

## Figures and Tables

**Table 1:** Relevant structural and ground-state magnetic properties of the Heusler compounds investigated in this work.

		NiMnSb		Co_2_MnSi		Mn_3_Ga		Ni_2_MnGa		Co_2_FeSi	
Structural phase		C1_*b*_		L2_1_		D0_22_		L2_1_		L2_1_	
Lattice parameters (*Å*)		*a* = 5.90		*a* = 5.64		*a* = 3.77*c* = 7.16		*a* = 5.81		*a* = 5.64	
Local Moments (*μ*_*B*_)	X	Ni	+0.30	Co	+1.05	Mn(2,3)	+2.01	Ni	+0.37	Co	+1.23
	Y	Mn	+3.62	Mn	+2.91	Mn(1)	−2.46	Mn	+3.14	Fe	+2.65
	Z	Sb	−0.05	Si	−0.04	Ga	−0.02	Ga	−0.02	Si	−0.01
Total Moment/atom (*μ*_*B*_)		1.33		1.25		0.04		1.02		1.26	

All calculations are performed using the local density approximation for exchange-correlation functional.
